# Application of a complex Si–Al–Fe reducing agent for the production of a nickel-containing alloy

**DOI:** 10.1038/s41598-026-45605-y

**Published:** 2026-03-24

**Authors:** Dauren Yessengaliyev, Bauyrzhan Kelamanov, Oleg Zayakin, Otegen Sariyev, Lyudmila Mikhailova, Talgat Zhuniskaliyev, Yerbol Kuatbay, Nurzhan Nurgali, Gulnur Abikenova, Assylbek Abdirashit, Aigerim Abilberikova

**Affiliations:** 1 Department of Metallurgy and Mining, K. Zhubanov Aktobe Regional University, Aktobe, 030000 Kazakhstan; 2 Vatolin institute of metallurgy of the Ural Branch of the Russian Academy of Sciences, Ekaterinburg, 620016 Russia; 3 Department of Science, Eurasian Technological University, Almaty, 050000 Kazakhstan; 4 Department of Metallurgy and Materials Science, Karaganda Industrial University, Temirtau, 101400 Kazakhstan; 5 ERG Research and Engineering Center, Astana, 010000 Kazakhstan

**Keywords:** Lateritic nickel ore, Metallothermy, Ferrosilicoaluminum, Ferrosilicon, Aluminum slag, Thermodynamic modeling, Rotatable central composite design, Nickel-containing alloy, Chemistry, Engineering, Environmental sciences, Materials science

## Abstract

To address the significant environmental challenges and technological limitations of conventional carbothermic ferronickel production, this study presents and optimizes an innovative metallothermic smelting process employing a complex silicon–aluminum–iron reducing agent (ferrosilicoaluminum, FeSiAl). For the first time, a comprehensive methodology integrating thermodynamic analysis, kinetic modeling, experimental design, and pilot-scale validation smelting has been applied to optimize the production of a nickel-containing alloy from lateritic ores of the Batamsha deposit (Kazakhstan). Thermodynamic modeling (HSC Chemistry) demonstrated that the combined use of Si and Al creates more favorable conditions for NiO reduction compared with their separate application, as evidenced by more negative ΔG values and higher equilibrium constants over the investigated temperature range (100–1600 °C). Kinetic analysis based on non-isothermal thermogravimetric and differential thermal analysis (TG-DTA) revealed a pronounced synergistic effect: the FeSiAl system exhibits the lowest apparent activation energy (16.15 kJ mol^−1^, which is 57% and 68% lower than those for ferrosilicon and aluminum-containing slag, respectively. This indicates a substantially enhanced reducibility and lower kinetic limitations. Process optimization was achieved through thermodynamic modeling in FactSage combined with a second-order rotatable central composite design (CCD). This approach enabled the development of predictive response surface models and the determination of optimal process parameters: smelting temperature of 1300–1350 °C, FeSiAl addition of 10 wt%, and lime flux addition of 38–40 wt%. Validation smelting experiments conducted in a laboratory ore-thermal electric furnace confirmed the accuracy of the model, yielding 9.5 kg of a multicomponent alloy with the following composition (wt%): Fe 70.0, Ni 8.0, Si 17.0, Cr 3.5, and Al 0.8. The accompanying slag exhibited a technologically favorable composition (wt%): SiO_2_ 48.6, CaO 36.4, Al_2_O_3_ 10.2, and MgO 4.5, with a very low residual nickel oxide content (NiO 0.1%), confirming the high reduction efficiency. The recovery rates of iron and chromium into the metallic phase were 71% and 83%, respectively. The resulting Fe-Ni-Si-Cr-Al alloy is proposed as a potential master alloy for steelmaking or as a reducing agent in metallurgical processes. The developed FeSiAl-based metallothermic process represents an energy-efficient and environmentally more sustainable alternative to conventional carbothermic technology.

## Introduction

The modern development of the metallurgical industry is closely associated with the growing demand for nickel- and chromium-containing alloys, which are widely used in the production of stainless steels as well as heat-resistant and corrosion-resistant materials. The increasing demand for these alloys necessitates the improvement of production technologies and the reduction of manufacturing costs.

Conventional technologies for producing nickel-containing ferroalloys, particularly ferronickel, are largely based on the carbothermic reduction of lateritic nickel ores. Despite their widespread industrial application, these processes are accompanied by a number of significant environmental and technological limitations. The use of carbon-based reducing agents (coke, anthracite, coal) results in substantial carbon dioxide emissions: up to 4–5 t of CO_2_ equivalent per ton of nickel produced^1,2^. In addition to reduction reactions, carbothermic systems generate sulfur oxides and other volatile compounds, which significantly complicate the operation of gas-cleaning systems and exert adverse environmental impacts^3^. Carbothermic reduction of nickel and iron proceeds predominantly according to the following reactions:

**Table:** 

NiO + C→ Ni + CO↑	ΔGº= −106,0 kJ·mol^− 1^, at T = 673,15 K
3Fe_2_O_3_ + C→ 2Fe_3_O_4_ + CO↑	ΔGº= −149,3 kJ·mol^− 1^, at T = 673,15 K
Fe_3_O_4_ + C→ 3FeO + CO↑	ΔGº= −46,7 kJ·mol^− 1^ at T = 1073,15 K
FeO + C→ Fe + CO↑	ΔGº= −24,3 kJ·mol^− 1^ at T = 1173,15 K

These reactions are accompanied by the formation of a significant amount of gaseous products (CO, CO_2_), which deteriorates furnace gas dynamics at elevated FeO concentrations in the oxide melt and promotes the entrainment of fine ore particles by the gas stream. The thermodynamic efficiency of carbon as a reducing agent also has certain limitations: despite the negative values of the standard Gibbs free energy change (ΔG°) for reduction reactions at temperatures above 1000 °C, the presence of kinetic barriers and the complex mineralogical composition of lateritic ores necessitate operation at elevated temperatures (T > 1350 °C). This results in increased energy consumption and reduced reduction selectivity^4,5^. An additional drawback of the carbothermic process is the potential formation of carbide phases (Fe_3_C, Ni_3_C), which alters the phase composition of the alloy, thereby complicating the refining process and potentially leading to instability in the properties of the produced ferronickel^6^.

The declining quality of lateritic nickel ores, together with increasingly stringent environmental requirements for metallurgical production, is making traditional carbon-based reducing agents progressively less effective^7^. The mineralogical and phase composition of lateritic nickel ores is characterized by a complex structure, in which nickel is often present in an isomorphic form within the crystal lattices of iron-bearing minerals such as goethite, olivine, and fayalite^8,9^. The presence of stable silicate and oxide phases results in a significant fraction of nickel being transferred to the slag phase during carbothermic reduction, thereby reducing its recovery into the metallic product^10,11^. In this context, the replacement of carbon-based reducing agents with alternative reducing systems represents a promising approach for the production of nickel-containing alloys and underscores the relevance of the present study^12–15^.

The aim of the present study is to produce nickel-containing alloys from lateritic nickel ores by a metallothermic route and to determine the influence of charge composition and temperature on the recovery of elements into the smelting products using mathematical modeling.

As is well known, the metallothermic reduction process is based on the use of active metallic reducing agents (silicon and aluminum) that exhibit a high affinity for oxygen.

The study presents the results of thermodynamic modeling of the silicothermic reduction of nickel, which confirm the high efficiency of this method. In particular, for the multicomponent CaO-SiO_2_-MgO-Al_2_O_3_-FeO-NiO-P_2_O_5_ system at temperatures of approximately 1500 °C, it was shown that the use of silicon or ferrosilicon makes it possible to achieve a nickel reduction degree of 99.7–99.8%. The obtained results indicate the strong potential of the silicothermic approach for processing nickel-bearing raw materials. At the same time, a significant limitation of this method is emphasized, namely the intensive enrichment of the metallic phase with silicon, which may restrict the direct application of the produced alloys without additional refining^16–20^.

Alongside the silicothermic process, aluminothermic reduction of nickel, based on the use of aluminum or aluminum-based alloys as reducing agents, is of considerable interest. Aluminothermic reactions are characterized by a high thermal effect, which enables a self-sustaining smelting regime and efficient reduction of nickel and iron oxides. A number of studies have shown that the use of metallic aluminum or aluminum-containing non-ferrous metallurgical wastes in the processing of nickel-bearing slags and concentrates provides a metallic phase reduction degree of up to 90–95%. In addition, a decrease in carbon content in the product and improved metal–slag separation are observed. However, aluminothermic processes are associated with challenges related to heat balance control, necessitating optimization of the charge composition and the selection of rational temperature regimes^21^.

From a technological perspective, combining the properties of silicon and aluminum within a single reducing agent opens additional opportunities for controlling the course of metallothermic reactions. In this regard, the present study proposes the use of ferrosilicoaluminum as a reducing agent for smelting nickel-containing alloys. The application of this material makes it possible to integrate the advantages of both silicothermy and aluminothermy-ensuring a high degree of nickel reduction at moderate temperatures while reducing the content of undesirable impurities in the metallic phase.

## Materials

Two types of nickel ores from the Batamsha deposit (Republic of Kazakhstan, Western Region, Aktobe) were used as the ore feedstock. The chemical composition of these ores is presented in Table 1.

**Table 1:** Chemical composition of nickel ore, wt%.

Material	Total Fe	Total Ni	SiO_2_	Al_2_O_3_	MgO	Cr_2_O_3_
Sample 1	20,50	1,18	50,50	4,50	3,66	3,92
Sample 2	10,80	0,82	55,80	12,50	7,70	1,84

As reducing agents for studying the metallothermic process, ferrosilicon, ferrosilicoaluminum, and aluminum slag were used. Their chemical compositions are presented in Table 2.

**Table 2:** Chemical composition of reducing agents, wt%.

Material	Fe	Si	Al	C	*P*
Ferrosilicon	29,87	70	–	0,08	0,05
Ferrosilicoaluminum	44,75	40	15	0,2	0,05
	Fe_2_O_3_	SiO_2_	Total Al	Al (metallic)	Na_2_O
Aluminum slag	0,58	0,64	57,90	17,84	2,59

Aluminum slag, a by-product of secondary aluminum processing, was chosen instead of pure aluminum to evaluate the feasibility of utilizing technogenic waste as a low-cost alternative. Its metallic aluminum content (17.84 wt%) is sufficient for reduction, and its use aligns with the goal of developing an economically viable and environmentally sustainable process.

As a fluxing material, lime was used throughout the entire experimental program. Its composition was as follows: CaO > 85%, CO₂ 0.67%, P 0.004%, and loss on ignition (LOI) 10%.

### Methods

**Fig. 1:**
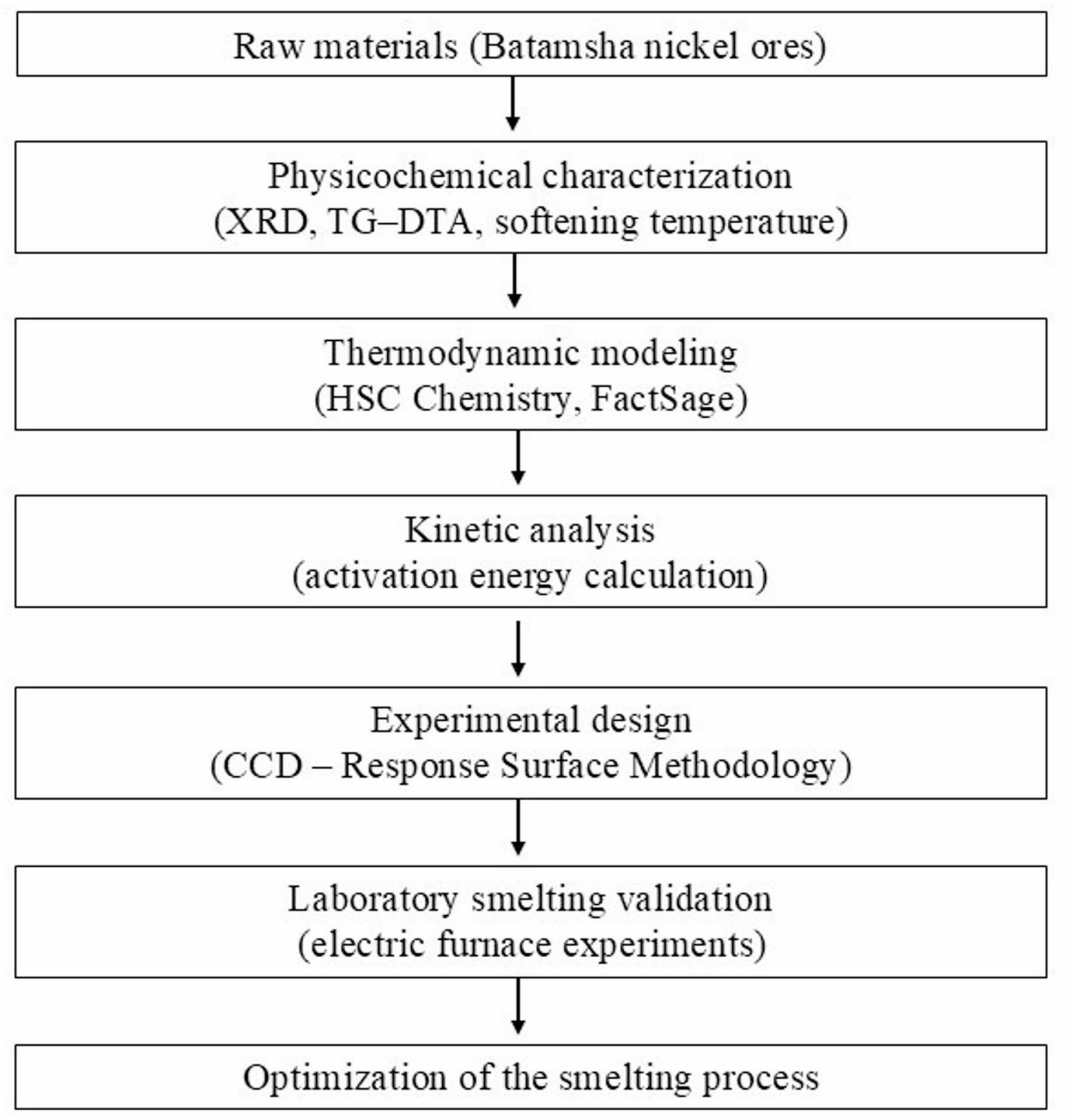
Overall methodological scheme of the study.

The overall methodological framework of the study is presented in Fig. 1.

#### Thermodynamic modeling

A thermodynamic analysis was carried out to theoretically assess the feasibility and efficiency of nickel reduction using various reducing agents. Calculations of the standard Gibbs free energy change (ΔG°) and the logarithm of the equilibrium constant (lg Kp) for the reactions of nickel oxide reduction by silicon, aluminum, and their combination (Si + Al) were performed using the HSC Chemistry 10.0 software package (Metso Outotec, Finland; https://www.hsc-chemistry.com) with the Reaction Equations module. The calculations were conducted over a temperature range of 100–1600 °C^22^.

Thermodynamic calculations of the multicomponent system and optimization of process parameters were performed using the FactSage 8.2 software package (Thermfact/CRCT, Canada and GTT-Technologies, Germany; https://www.factsage.com) Equilibrium composition calculations were carried out using the *Equilib* module. The thermodynamic databases *FactPS* and *FToxid* were used to describe the gas, metal, and oxide phases, enabling the consideration of non-ideal behavior of oxide melts. The modeling aimed to determine the equilibrium distribution of elements between the metallic and slag phases as a function of temperature and charge composition.

#### Physicochemical analysis of raw materials

The chemical composition of the nickel ores and reducing agents was determined by classical wet chemical analysis following the relevant GOST standards: GOST 22772.4–96 (for Fe and Ni), GOST 22772.2–96 (for SiO_2_), GOST 22772.3–96 (for Al_2_O_3_ and MgO), and GOST 22772.5–96 (for Cr_2_O_3_)^23–26^. Additionally, a control analysis was performed using an emission spectrometer DFS-500D equipped with an MFS-12 oxide attachment. The instrument was calibrated with certified reference materials (GSО 1762-93 for nickel ore and GSO 2296−82 for ferroalloys)^27,28^. The relative uncertainty for major components (Fe, Ni, SiO_2_) did not exceed ± 2–3%, and for minor components (Cr_2_O_3_, MgO, Al_2_O_3_) it was ± 4–5%. Accuracy was verified by triplicate measurements and comparison with certified values.

The phase composition of the nickel ores was investigated using a Bruker D8 ADVANCE X-ray diffractometer (Cu Kα radiation, λ = 1.5406 Å). The measurement conditions were as follows: accelerating voltage of 40 kV, current of 40 mA, scanning range of 2θ = 10–90° with a step size of 0.02°, and a scanning speed of 1°·min⁻¹. Prior to analysis, the samples were ground to a particle size of < 63 μm and dried at 105 °C.

To study the thermal behavior of the ore and its mixtures with reducing agents, a NETZSCH STA 449 F3 Jupiter simultaneous thermal analyzer was used. The experiments were carried out in an argon atmosphere at a heating rate of 15 °C·min⁻¹, with a sample mass of 0.15 g. Additional investigations of mixtures containing aluminum slag were performed using a NETZSCH STA 409 PC/PG analyzer in a nitrogen atmosphere up to a temperature of 900 °C.

**Fig. 2:**
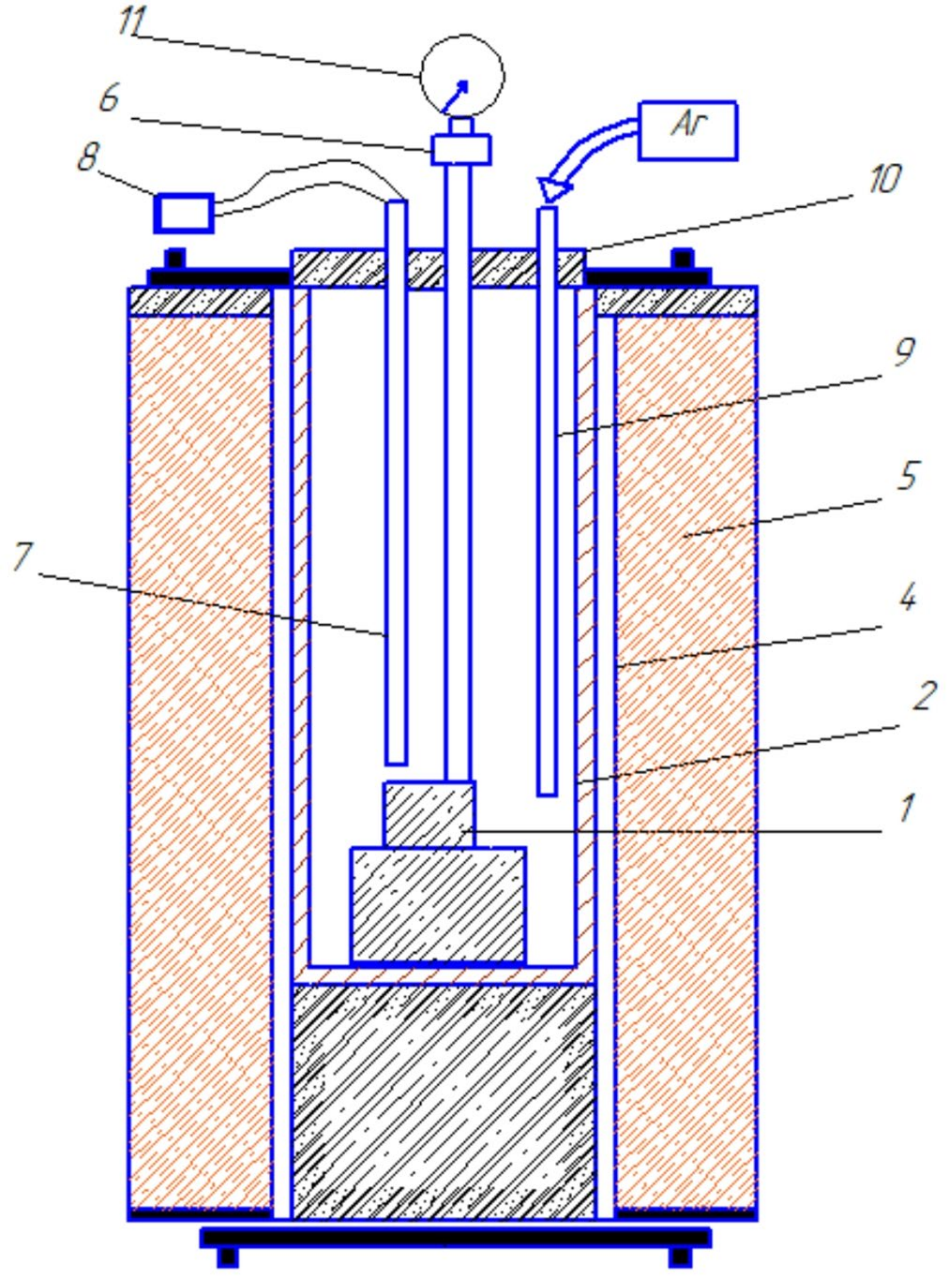
Setup for determining the softening onset temperature and the softening temperature interval of ores. (**1**) Crucible with sample; (**2**) Protective alumina cap; (**3**) Graphite heater; (**4**) Tammann furnace; (**5**) Rod with foot; (**6**) Load; (**7**) Thermocouple; (**8**) Voltmeter; (**9**) Alumina tube for inert gas supply; (**10**) Insulating lid; (**11**) Shrinkage indicator.

The determination of the softening onset temperature and the softening temperature interval of the ores was performed as follows. The particle size of the nickel ore was 3–5 mm; after preliminary drying at 105 °C, the material was loaded into cylindrical alumina crucibles. The sample in the crucible was compacted, and the height of the compacted sample layer was (50 ± 1) mm. A schematic diagram of the setup used to determine the softening onset temperature and the softening temperature interval is shown in Fig. 2.

The mass of the load was calculated so that the pressure applied to the sample was 0.1 MPa. After installing the required equipment, furnace heating was initiated, while an inert gas was simultaneously introduced into the heating chamber. When the chamber temperature reached 700 °C, the indicator recording the movement of the rod was set to the zero position. The tests were conducted at a heating rate of 10 °C·min⁻¹, and the indicator readings were recorded as a function of temperature. Taking into account the natural thermal expansion of the rod during heating, the shrinkage coefficient of the charge was calculated. The softening onset temperature was defined as the temperature at which the rod penetrated 1% into the sample, while the softening end temperature was defined as the temperature at which the rod penetrated 40% of the initial height of the compacted sample layer, in accordance with GOST 26517–85^29^.

#### Kinetic analysis

To quantitatively evaluate the kinetics of thermal transformations based on DTA data, the apparent activation energy (Ea) for the key stages was calculated. The calculations were performed using a method based on the Arrhenius equation at a constant heating rate of β = 15 °C·min^− 1^. It was assumed that the deviation of the DTA signal (Δt) is proportional to the reaction rate. The activation energy was determined from the slope of the linear approximation of the dependence lg(Δt) = f(1/T), constructed for the characteristic peaks on the thermograms.

It should be noted that the single-heating-rate method (a simplified Kissinger approach) yields approximate values and does not allow separation of chemical and diffusional contributions. However, for the purpose of this study – comparative evaluation of the relative efficiency of different reducing agents under identical experimental conditions – this approach is acceptable and widely used in preliminary technological assessments^30,31^. The obtained Ea values are therefore treated as comparative indicators rather than absolute kinetic constants.

#### Experimental design and optimization

To systematically determine the optimal parameters of the nickel-containing alloy smelting process using FeSiAl, a design of experiments (DoE) approach was applied. A second-order rotatable central composite design (CCD) was employed for three factors: X_1_ – process temperature (°C), X_2_ – mass fraction of the FeSiAl reducing agent in the charge (wt%), and X_3_ – mass fraction of lime in the charge (wt%).

The center point of the design (zero level) corresponded to the following values: temperature of 1300 °C, FeSiAl content of 20 wt%, and lime content of 30 wt%. The experimental design comprised 20 runs (8 factorial points, 6 axial points, and 6 replicated center-point runs to estimate the reproducibility variance). Within each virtual experiment, thermodynamic modeling was performed in the Equilib module (FactSage) to calculate the response variables: Y_1_ – degree of iron reduction (ηFe, %), Y_2_ – degree of chromium reduction (ηCr, %), and Y_3_ – silicon content in the metallic phase (CSi, wt%).

The obtained data were processed using regression analysis to construct second-order polynomial models and corresponding response surface plots. The regression analysis and construction of three-dimensional response surfaces were performed using PTC Mathcad Prime 9.0 (Parametric Technology Corporation, USA; https://www.ptc.com).

#### Large-scale laboratory smelting (validation experiment)

To verify the results of modeling and optimization, laboratory smelting experiments were carried out. The experiments were performed in a single-phase ore-thermal electric furnace with a transformer power of 100 kVA, equipped with a graphite electrode 100 mm in diameter. Smelting was conducted at a secondary voltage of 18 V and a current of 80–100 A on the primary winding.

The smelting procedure included the following steps:

1. Preheating of the furnace for 120 min using 0.2 kg of coke fines as a current-conducting bed.
2. Cleaning of the furnace bath from residual coke and charging of 0.5 kg of ore to form the initial melt.
3. Layer-by-layer charging of the main charge (particle size 5–10 mm). The charge composition was varied according to the validation objectives.
4. Operation of the process with periodic (every 2 h) tapping of metal and slag through the tap hole. The temperature regime was monitored indirectly based on the melting behavior of the burden layer and the stability of the electrical operating conditions.

The metallic ingots and slag samples obtained after smelting were subjected to chemical analysis to determine their elemental composition and to calculate the recovery coefficients of elements into the alloy.

## Results and discussion

### Thermodynamic analysis of nickel reduction

**Fig. 3:**
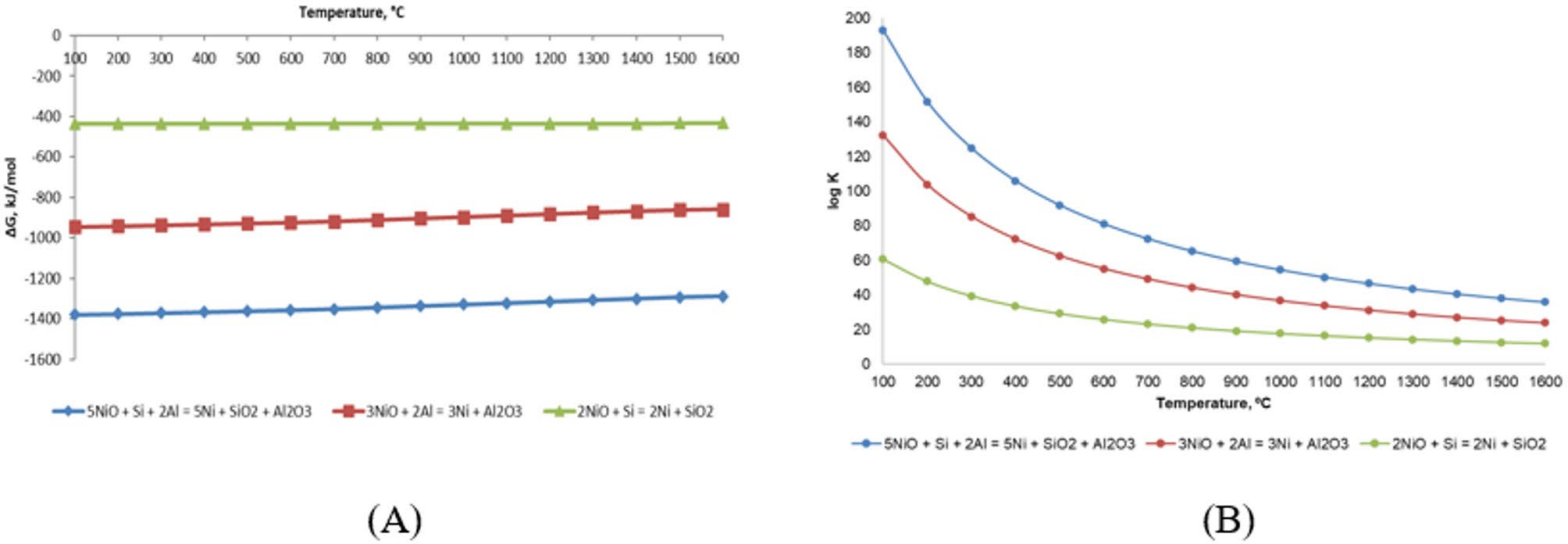
Temperature dependences of thermodynamic parameters of nickel oxide reduction reactions in the NiO-Al, NiO-Si, and NiO-Si-Al systems: (**A**) Gibbs free energy; (**B**) Logarithm of the equilibrium constant.

As a theoretical basis for selecting an appropriate reducing agent, a thermodynamic analysis of nickel oxide reduction reactions was performed. Figure 3 presents the temperature dependences of the Gibbs free energy change (ΔG) and the logarithm of the equilibrium constant (lg Kp), calculated using HSC Chemistry, for three systems: NiO-Al (aluminothermy), NiO-Si (silicothermy), and NiO-Si-Al (combined reduction).

Based on the calculations, the temperature dependences of the Gibbs free energy change (ΔG) and the logarithm of the equilibrium constant (lg Kp) were determined over the temperature range of 100–1600 °C for the following reactions:

2NiO + Si→ 2Ni + SiO_2_;

3NiO + 2Al → 3Ni + Al_2_O_3_;

5NiO + Si + 2Al → 5Ni + SiO_2_ + Al_2_O_3_.

The obtained dependences (Fig. 1) show that, in all the systems considered, the Gibbs free energy change (ΔG) remains negative over the entire temperature range, indicating the thermodynamic feasibility of nickel oxide reduction reactions. At the same time, the lowest ΔG values are observed for the combined NiO-Si-Al system, demonstrating its highest thermodynamic favorability compared with the individual aluminothermic and silicothermic processes. Consequently, the use of a complex reducing agent containing both aluminum and silicon is the most preferable approach for the deep reduction of nickel from oxide compounds.

### Physicochemical characterization of nickel ores

#### Mineralogical composition (XRD)

The performance of ferroalloy smelting units and the technical and economic indicators of their operation depend strongly on the quality and properties of the feed ores and their preparation for smelting. Among the key factors affecting furnace operation are the softening onset temperature of the ores and the softening temperature interval, as well as their thermal behavior, which together determine the conditions for reduction reactions, the degree of element recovery into the alloy, and the temperature regime in the reaction zone of ore-reduction electric furnaces^32^. In the present study, these properties are systematically examined with respect to nickel ores from the Batamsha deposit.

The softening onset temperature and the softening temperature interval are key parameters for classifying ores with respect to their reducibility. For example, ores exhibiting a high softening onset temperature and a narrow softening interval are classified as easily reducible when processed in ore-thermal furnaces. In contrast, difficult-to-reduce ores are characterized by low softening onset temperatures and a wide softening interval. During the smelting of easily reducible ores in ore-reduction electric furnaces, primary slags are formed in the lower furnace zones, whereas in the case of difficult-to-reduce ores, slag formation occurs in higher furnace zones, which complicates gas-dynamic conditions.

The softening onset temperature of nickel ores is largely determined by their mineralogical composition and the contact area between particles composed of different minerals. In this regard, phase composition studies of two nickel ore samples were carried out using X-ray diffraction (XRD) analysis. The XRD patterns of the investigated nickel ore samples are presented in Fig. 3.

**Fig. 4:**
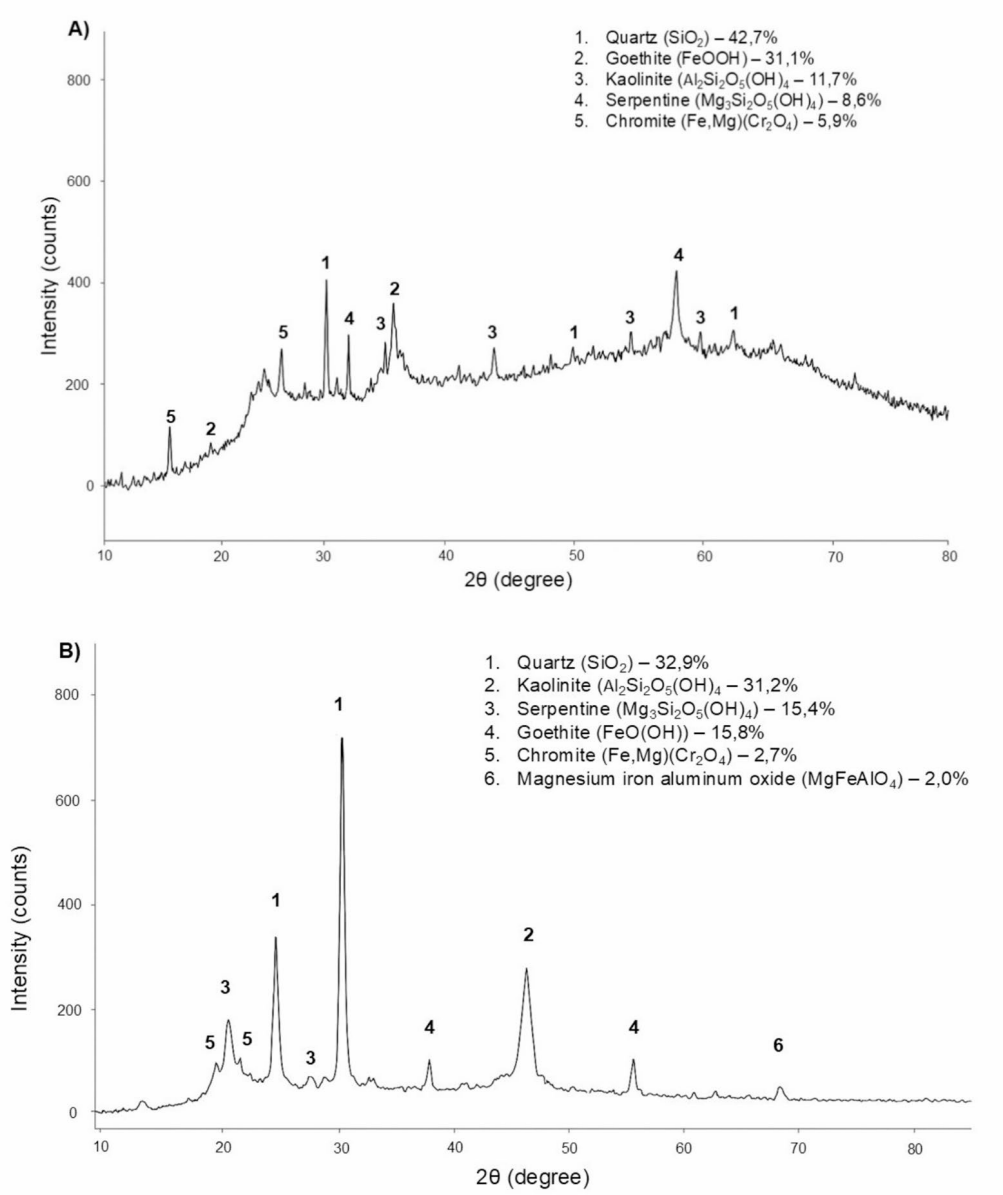
X-ray diffraction patterns of nickel ores: (**A**) Sample 1; (**B**) Sample 2.

The X-ray diffraction analysis (Fig. 4) revealed significant differences in the mineralogical composition of the two ore samples from the Batamsha deposit, which explain the differences observed in their chemical compositions (Table 1).

According to the X-ray diffraction analysis, Sample 1 is characterized by quartz (SiO₂), goethite (FeO(OH)), serpentine (Mg_3_Si_2_O_5_(OH)_4_), and chromite ((Fe, Mg)Cr_2_O_4_) as the main crystalline phases. The predominance of quartz together with a well-developed assemblage of iron and aluminum hydroxide phases reflects a high degree of weathering of the original ultrabasic substrate. The sample also contains kaolinite and serpentine, which form a clay–silicate matrix of the ore. The presence of spinel-type chromite confirms that chromium occurs in a stable spinel phase. Overall, the phase composition of Sample 1 is dominated by silicon- and iron-bearing minerals with a moderate proportion of clay–silicate phases, which is typical of lateritic nickel ores.

X-ray diffraction analysis of Sample 2 showed that the major phases are quartz (SiO_2_), kaolinite (Al_2_Si_2_O_5_(OH)_4_), serpentine (Mg_3_Si_2_O_5_(OH)_4_), goethite (FeO(OH)), and chromite ((Fe, Mg)Cr_2_O_4_)ж in addition, minor amounts of magnesium–iron–aluminum spinel (MgFeAlO_4_) were detected. Sample 2 is characterized by a more complex mineralogical composition with a higher content of aluminosilicate and clay phases. Quartz and kaolinite are the most abundant phases, reflecting the increased Al_2_O_3_ content and indicating a well-developed clay component in the ore. Minor amounts of chromite and spinel indicate the presence of chromium- and aluminum-bearing spinel phases. Compared with Sample 1, Sample 2 exhibits a higher proportion of aluminosilicate and magnesium-bearing minerals and a lower fraction of iron-bearing phases.

X-ray diffraction analysis did not reveal any discrete nickel-bearing phases in the investigated samples, which is attributed to the low nickel content and the specific mineralogical distribution of nickel in lateritic ores.

In these ores, nickel is predominantly present in the form of isomorphic substitution within iron- and magnesium-bearing minerals. The primary host of nickel is likely goethite (FeO(OH)), in which Ni^2+^ ions substitute for Fe^3+^ in octahedral positions. Such substitution is typical of oxidized lateritic ores and does not result in the formation of a separate crystalline nickel phase detectable by XRD. Additional nickel may be associated with serpentine (Mg_3_Si_2_O_5_(OH)_4_), where Ni^2+^ substitutes for Mg^2+^ in the crystal lattice of magnesium silicates.

At the next stage of the study, changes in the characteristics of the ore samples during heating were investigated.

#### Thermal behavior and softening temperature

**Fig. 5:**
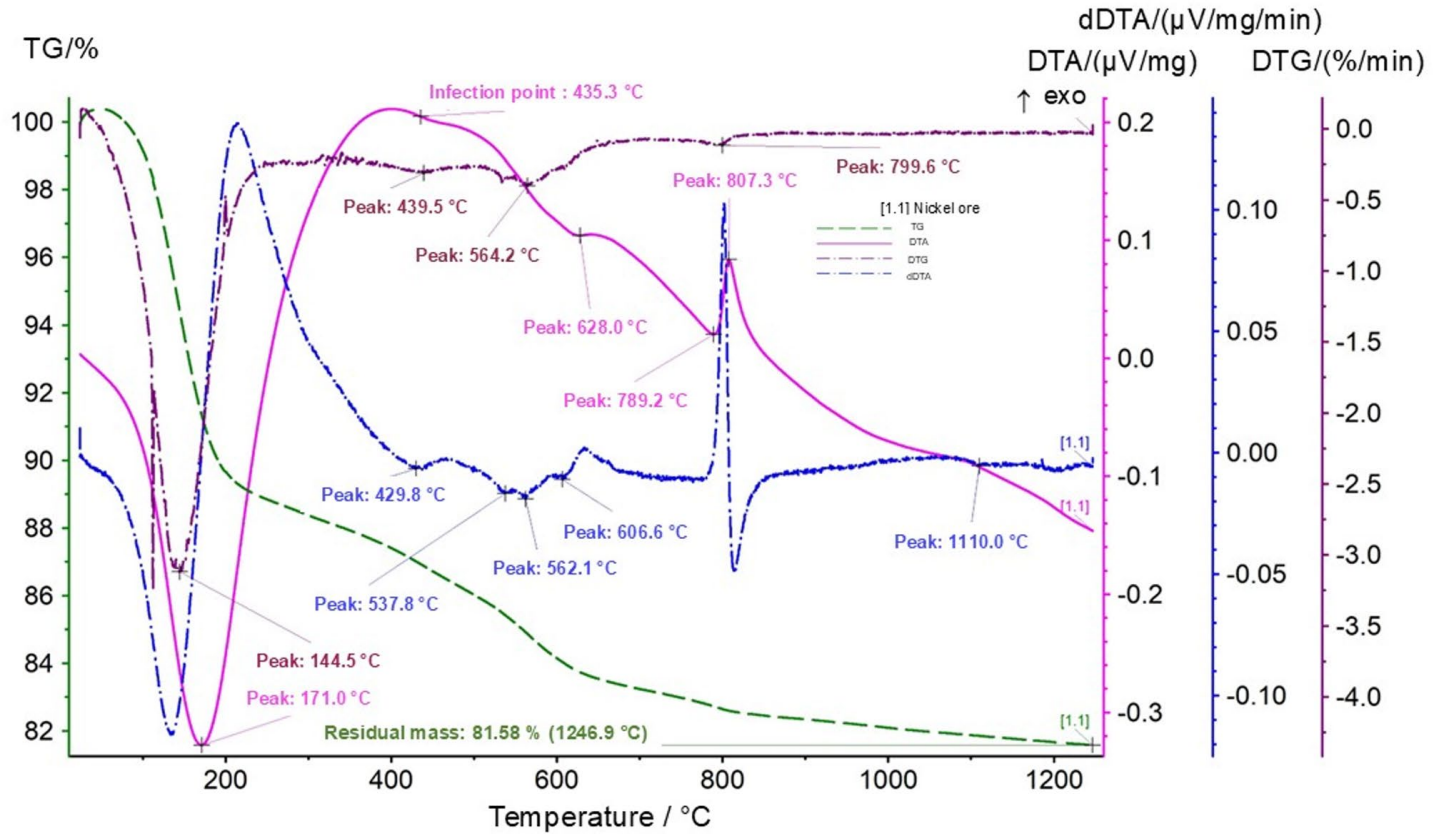
Thermogram (TG, DTG, DTA) of nickel ore (Sample 1) TG (mass change, %) – green dashed line; DTG (mass change rate, %·min^− 1^) – burgundy dash–dot line; DTA (thermal effects, µV·mg^− 1^) – pink solid line; dDTA (µV·mg^− 1^·min^− 1^) – dark blue dash-dot line.

The thermogram of Sample 1 (Fig. 5) exhibits a series of characteristic effects, including endothermic peaks at approximately 171 °C (removal of adsorbed moisture) and 628–789 °C (dehydroxylation of silicates and hydroxides), as well as an exothermic peak at around 807 °C corresponding to the crystallization of new phases. These transformations directly affect the behavior of the ore during furnace processing.

The DTA curve exhibits the following characteristic thermal effects: an intense endothermic peak at 171 °C, an inflection of the curve at 435.3 °C, an endothermic effect with maximum intensity at 628 °C, an endothermic effect at 789 °C, and an exothermic peak at 807.3 °C. On the dDTA curve, additional weak endothermic extrema are observed at 537.8 °C, 562.1 °C, and 1110 °C.

The combination of a pronounced endothermic effect at 171 °C (DTA curve) and an endothermic extremum at 562.1 °C (dDTA curve) is interpreted as the manifestation of the clay mineral nontronite, (Ca, Na)Fe_2_^3+^[(Si, Al)_4_O_10_](OH)_2_ nH_2_O. The first endothermic effect corresponds to the removal of molecularly bound water, whereas the second reflects dehydroxylation and the release of structural (constitutional) water.

The inflection of the DTA curve at 435.3 °C occurs concurrently with a decrease in sample mass; on the DTG curve, this corresponds to a minimum at 439.6 °C. This effect is associated with the dehydration of goethite (α-FeOOH) followed by the formation of hematite (α-Fe_2_O_3_).

The combination of an endothermic effect at 789 °C followed by an exothermic peak at 807.3 °C is characteristic of the serpentine group of minerals, represented by the compound Mg_6_[Si_4_O_10_](OH)_8_. The endothermic effect corresponds to the breakdown of the serpentine structure and the removal of hydroxyl groups, whereas the exothermic effect reflects the crystallization of newly formed phases.

The simultaneous occurrence of the endothermic peak at 789 °C, the exothermic effect at 807.3 °C, and the endothermic peak at 628 °C may be associated with the presence of magnesium–nickel hydrosilicate, namely kerolite, (Mg, Ni)_3_Si_4_O_10_(OH)_2_·H_2_O.

The weak endothermic extremum at 537.8 °C observed on the dDTA curve may be attributed to the presence of an iron-rich chlorite impurity with the composition 2SiO_2_·Al_2_O_3_·(FeO)·H_2_O. The combination of the endothermic effect at 628 °C (DTA) and the endothermic extremum at 1110 °C (dDTA) is interpreted as a possible manifestation of the mineral revdinskite, 3(Ni, Mg)O·2SiO_2_·2H_2_O.

**Fig. 6:**
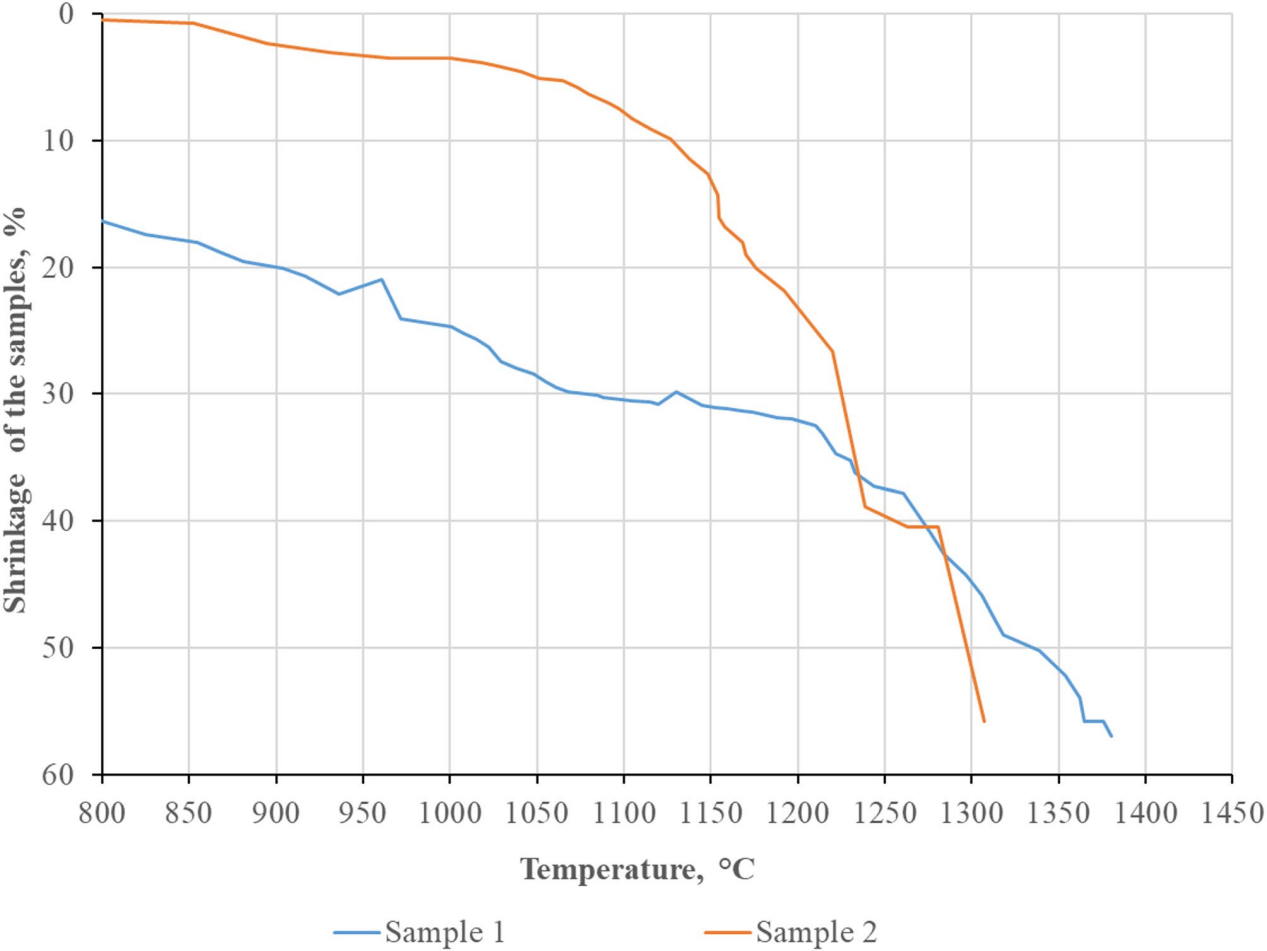
Shrinkage curves of nickel ores as a function of temperature.

From a technological standpoint, the softening temperatures are key parameters governing the formation of the burden column in the electric furnace. The data obtained in accordance with GOST 26517–85 are presented in Table 3; Fig. 6.

**Table 3:** Softening onset temperatures, softening temperature intervals, and softening end temperatures of nickel ores.

Sample No.	Softening onset temperature, °C	Softening temperature interval, °C	Softening end temperature, °C
1	773	603	1376
2	895	386	1281

According to the data in Table 3, Sample 1 is characterized by a lower softening onset temperature (773 °C) compared with Sample 2 (895 °C), indicating earlier development of structural transformations and lower thermal stability of Sample 1. At the same time, the softening temperature interval of Sample 1 is significantly wider (603 °C versus 386 °C for Sample 2), reflecting a more multistage nature of structural breakdown accompanied by a gradual decrease in mechanical strength. The softening end temperature for Sample 1 reaches 1376 °C, whereas Sample 2 becomes fully softened at 1281 °C.

Comparison of these data with the results of phase analysis allows Sample 1 to be considered more representative for subsequent analysis of high-temperature processes.

**Fig. 7:**
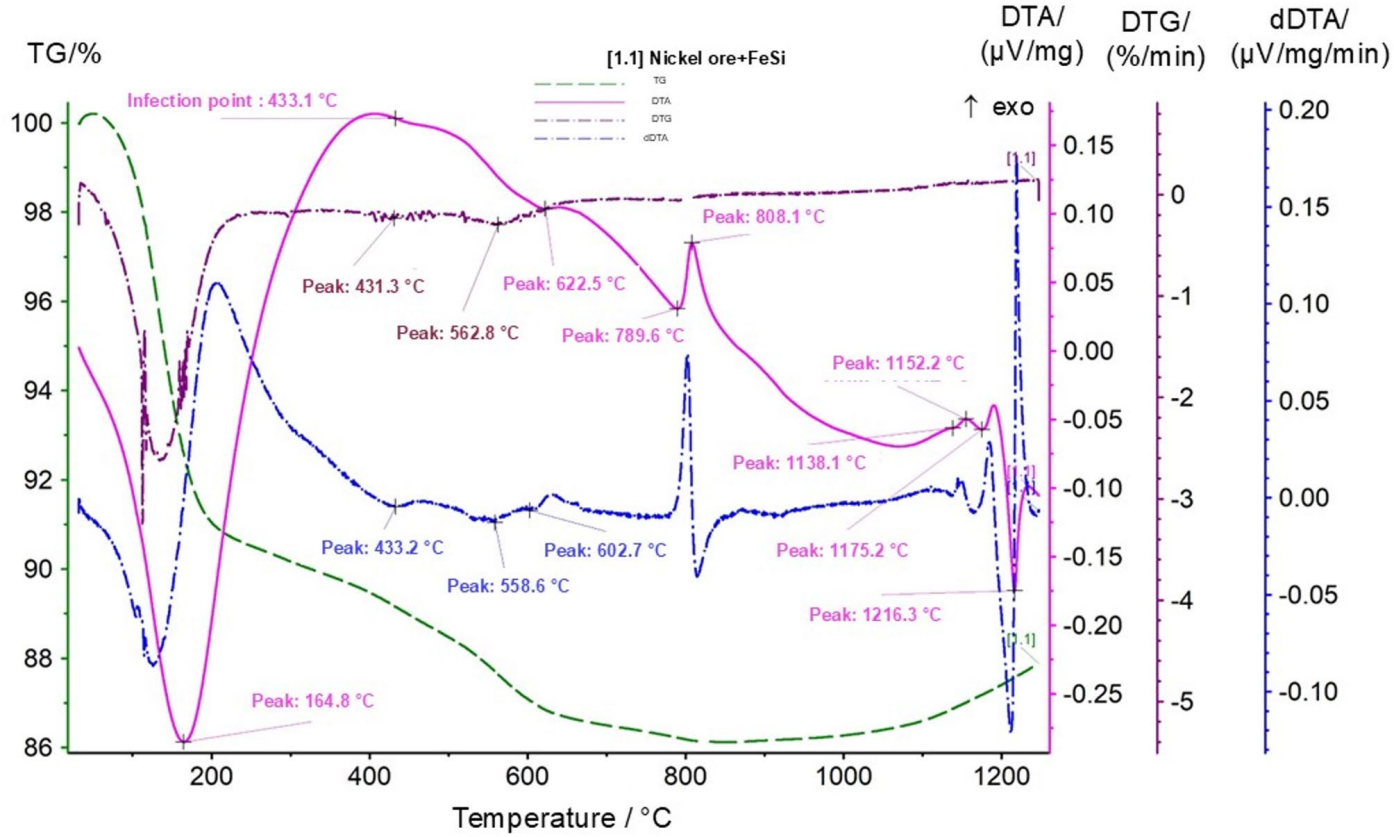
Thermogram of the mixture of nickel ore (Sample 1) with ferrosilicon.

**Fig. 8:**
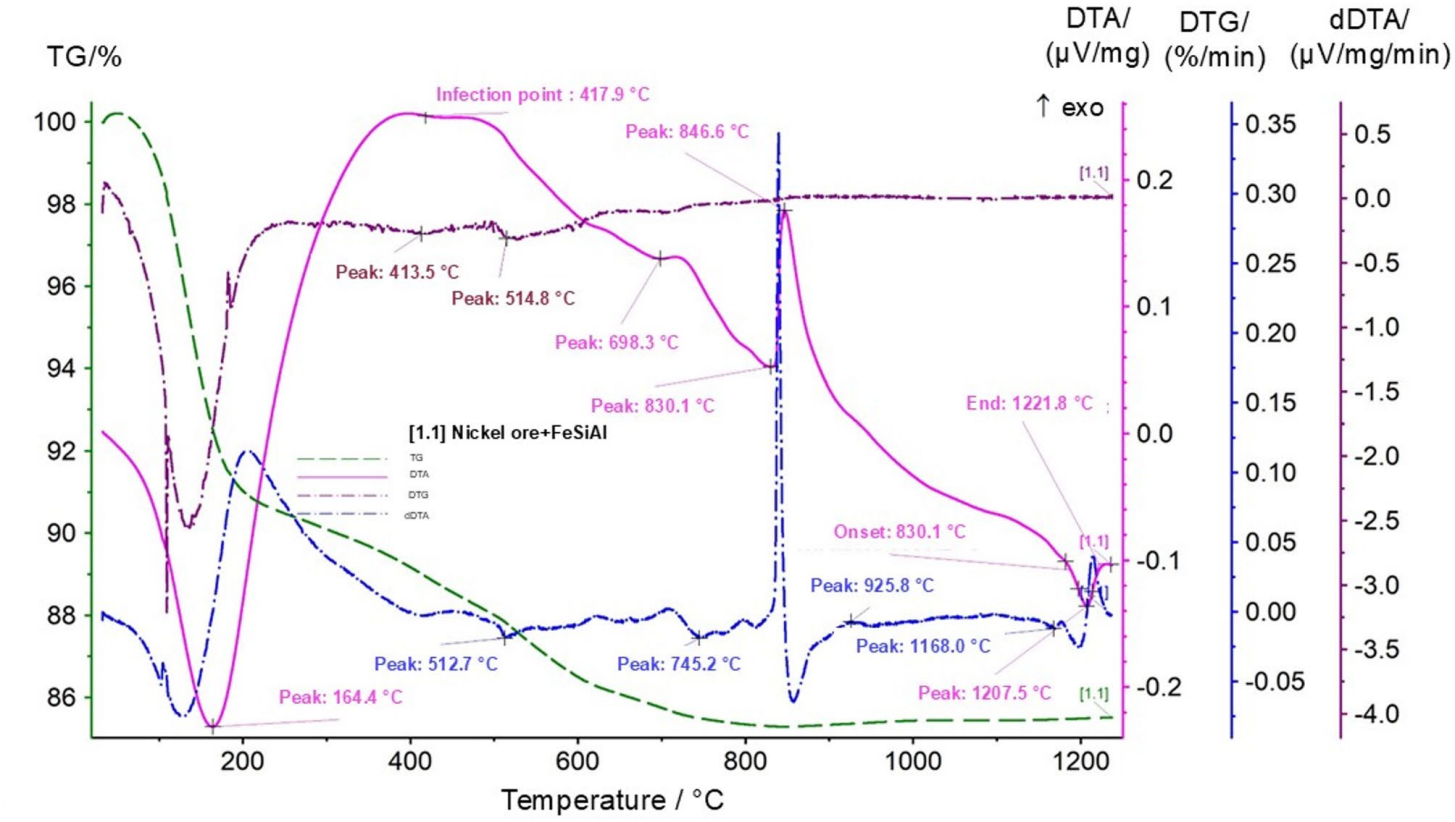
Thermogram of the mixture of nickel ore (Sample 1) with ferrosilicoaluminum.

Thermal analysis of ore mixtures with various reducing agents (FeSi, FeSiAl, aluminum slag) made it possible to assess the kinetics of the initial stages of the process. Figures 6, 7 and 8 present thermograms of the mixtures, demonstrating shifts and changes in the intensity of exothermic effects corresponding to reduction reactions.

As shown in Fig. 7, in the low-temperature region up to 200 °C, an endothermic effect associated with the removal of adsorbed moisture is observed. In the range of 400–450 °C, a pronounced endothermic peak appears, related to the decomposition of serpentine minerals and the release of structurally bound water, accompanied by a noticeable mass loss. In the temperature interval of 550–650 °C, weak effects are detected, associated with the decomposition of iron hydroxides and the restructuring of silicate phases. Exothermic peaks at 790–810 °C correspond to the crystallization of magnesium and iron silicates. The most intense exothermic processes observed in the range of 1130–1220 °C indicate metallothermic reduction of nickel and iron involving ferrosilicon and the onset of formation of a nickel-containing metallic melt.

The next stage of the study involved thermal analysis of a mixture of lateritic nickel ore with a ferrosilicoaluminum reducing agent. The results of the thermal analysis of the nickel ore–ferrosilicoaluminum mixture are presented in Fig. 8.

On the thermogravimetric (TG) curve, an initial mass loss is observed up to approximately 164 °C, which is attributed to dehydration and the removal of physically bound moisture. In the temperature range of 400–550 °C, a series of endothermic effects is observed on the DTA and DTG curves (peaks at 413.5 °C and 514.8 °C), associated with the decomposition of iron hydroxides and aluminosilicate phases of the ore.

In the range of 600–850 °C, pronounced thermal effects are detected (DTA peaks at 698.3 °C, 745.2 °C, 830.1 °C, and 846.6 °C), corresponding to phase transformations in the oxide–silicate matrix as well as the initial stages of interaction between nickel and iron oxides and the components of ferrosilicoaluminum. The peak at 417.9 °C is characterized by a noticeable inflection on the dDTA curve, indicating a change in the decomposition kinetics.

In the high-temperature region of 900–1200 °C, intense exothermic effects are recorded (DTA peaks at 925.8 °C, 1168.0 °C, and 1207.5 °C), reflecting active reduction of iron and nickel oxides by aluminum and silicon, as well as the formation of intermetallic and silicate phases. The temperature interval of 1189–1222 °C corresponds to the completion of the main reduction reactions and the formation of a liquid-phase melt, as confirmed by a characteristic decrease in the mass derivative and a pronounced exothermic minimum.

**Fig. 9:**
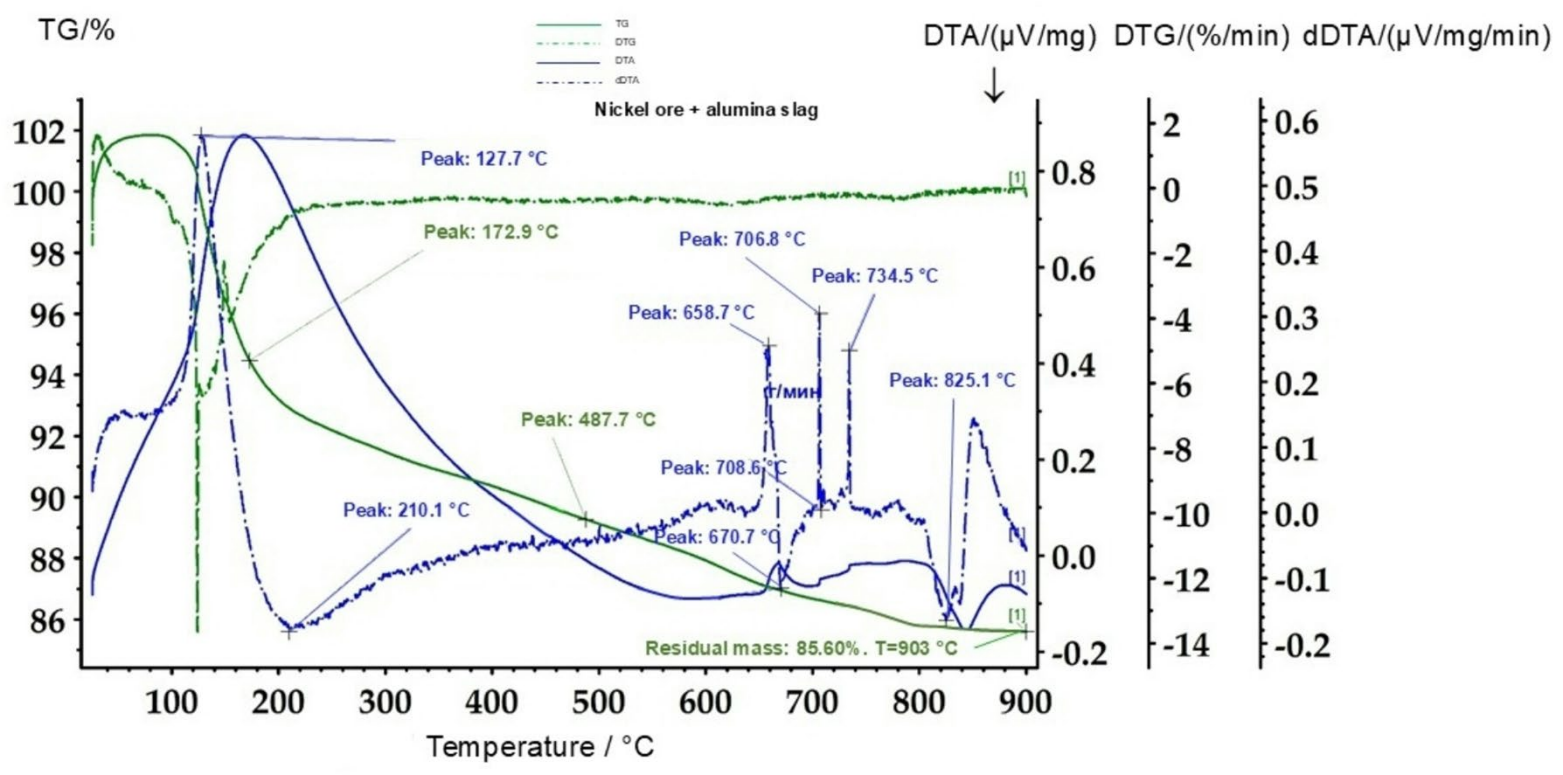
Thermogram of the mixture of nickel ore (Sample 1) with aluminum slag. TG (mass change, %)—green solid line; DTG (mass change rate, %·min^− 1^)—green dashed line; DTA (thermal effects, µV·mg^− 1^)—dark blue solid line; dDTA (µV·mg^− 1^·min^− 1^)—dark blue dash-dot line.

For further comparison of the thermal behavior of different reducing systems, the next stage involved thermal analysis of a mixture of nickel ore with aluminum slag. The results of the thermal analysis of the nickel ore–aluminum slag mixture are presented in Fig. 9.

As shown in Fig. 8, the thermal behavior of the nickel ore-aluminum slag mixture differs substantially from that observed in the previous test (nickel ore-ferrosilicon mixture) and is characterized by a different sequence of thermal effects. In the temperature range up to 200 °C, endothermic peaks at 127–173 °C are recorded, corresponding to the removal of adsorbed and weakly bound moisture. An exothermic effect at around 210 °C indicates the initial structural rearrangement of hydrated aluminosilicates present in the aluminum slag.

In the range of 450–500 °C, a pronounced endothermic effect (approximately 487 °C) is observed, associated with the decomposition of aluminum hydroxides and the transformation of amorphous aluminosilicates into more stable phases. Subsequent thermal effects in the interval of 650–735 °C are related to recrystallization of alumina-bearing phases and the interaction of ore oxides with components of the aluminum slag. High-temperature peaks (≈ 825 °C) reflect the formation of more complex aluminosilicate structures and the completion of degradation of hydrated minerals.

The overall mass loss to a residual value of approximately 85.6% at 900 °C indicates a significant proportion of hydroxide and volatile components in the mixture.

### Kinetics of thermal transformations and the synergistic effect of FeSiAl

**Fig. 10:**
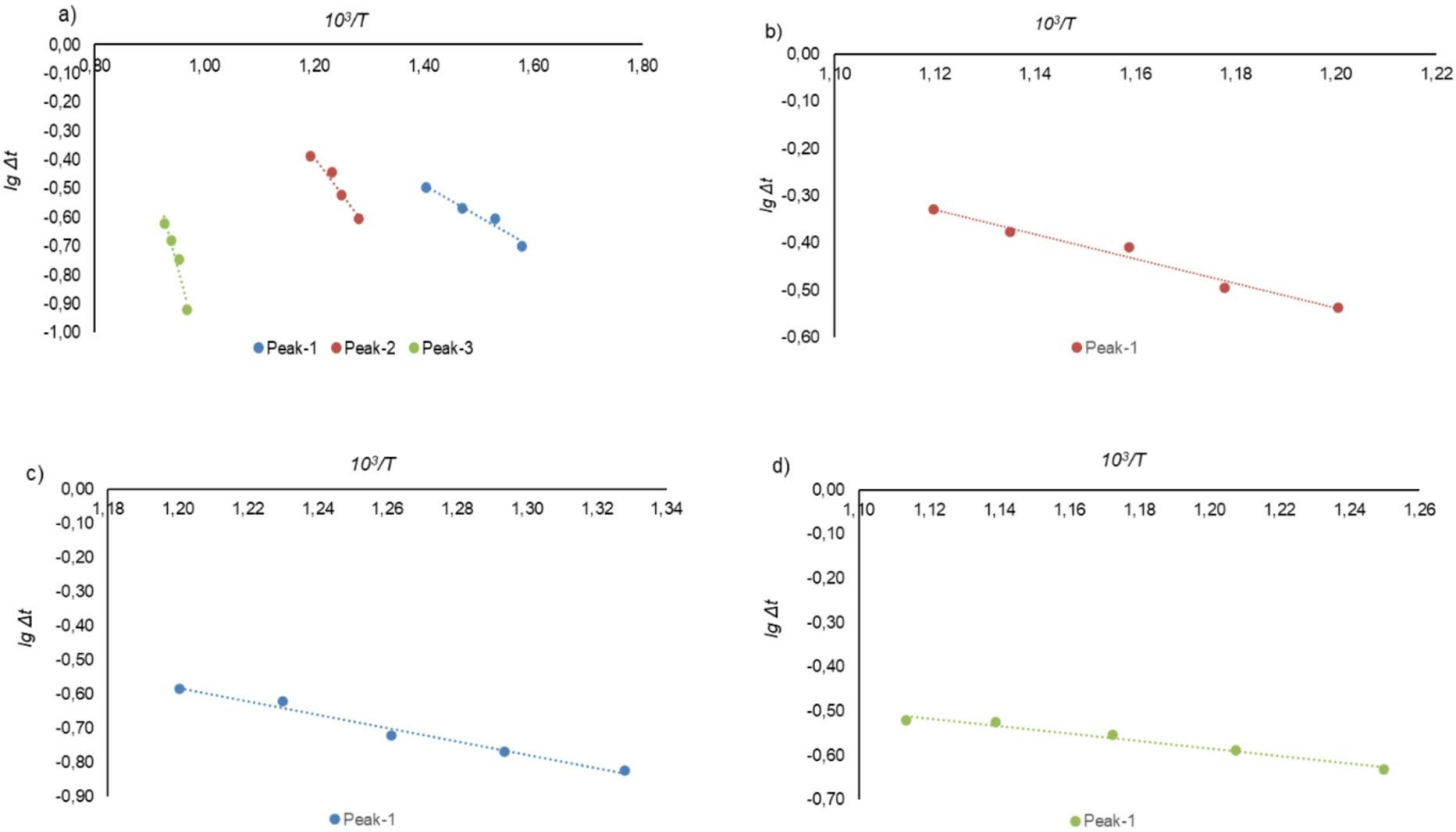
Dependence of the DTG peak height in logarithmic coordinates on the reciprocal temperature **a** nickel ore; **b** mixture of nickel ore with aluminum slag; **c** ixture of nickel ore with ferrosilicon; **d** mixture of nickel ore with ferrosilicoaluminum.

Thermal analysis of ore mixtures with different reducing agents revealed a shift in the exothermic effects associated with reduction reactions. Quantitative evidence of the enhanced reactivity of FeSiAl is provided by the calculated values of the apparent activation energy (Ea), which are summarized in Table 4 and visualized in the corresponding Fig. 10.

The activation energy (Ea) was evaluated from linear relationships plotted in the lg(Δt) – 10^3^/T coordinates, obtained for the characteristic thermal effects identified on the thermograms.

**Table 4:** Results of activation energy (Ea) calculations determined from the slope of the linear lg(Δt) − 10^3^/T dependence.

Material	Equation	Correlation coefficient (*R*)	Activation energy, Ea (kJ·mol^− 1^)	Temperature range, °C
Nickel ore	lgΔt = − 1087.566/T + 1.036	0.950	20.82	360–439
	lgΔt = − 2525.837/T + 2.643	0.960	48.36	507–564
	lgΔt = − 7225.52/T + 6.107	0.963	138.35	760–805
Nickel ore + aluminum slag	lgΔt = − 2621.61/T + 2.607	0.974	50.20	560–620
Nickel ore + ferrosilicon	lgΔt = − 1965.82/T + 1.778	0.977	37.64	480–560
Nickel ore + ferrosilicoaluminum	lgΔt = − 843.34/T + 0.428	0.975	16.15	527–625

The obtained value of apparent activation energy for the ore – FeSiAl system (16.15 kJ·mol^− 1^) is significantly lower than typical values for chemically controlled heterogeneous reactions (usually 50–300 kJ·mol^− 1^). Such a low Ea (below 20–25 kJ·mol^− 1^) is characteristic of processes limited by diffusion – either bulk diffusion in the melt or diffusion through a product layer^29,32^. This suggests that the combined presence of silicon and aluminum not only lowers the thermodynamic barrier but also promotes the formation of low-melting eutectics or modifies wetting, thereby accelerating mass transport of reactants to the reaction interface and removal of products. Thus, the low Ea reflects a predominantly diffusion-controlled mechanism in the FeSiAl system, which is kinetically advantageous because diffusion processes require lower energy input.

For the initial nickel ore, three characteristic temperature intervals corresponding to the main transformation stages were identified, within which the apparent activation energy (Ea) varies from 20.82 to 138.35 kJ·mol^− 1^ at temperatures of 360–439 °C, 507–564 °C, and 760–805 °C. The low Ea values characteristic of the initial temperature stages indicate the occurrence of hydroxide phase decomposition processes, whereas the increase in Ea with rising temperature reflects a transition to more energy-intensive transformations associated with modification and breakdown of the silicate structure of the ore.

A comparison of the kinetic parameters of different reducing systems shows that the lowest activation energy is characteristic of the nickel ore–ferrosilicoaluminum mixture (Ea = 16.15 kJ·mol^− 1^, T = 527–625 °C). For the “ore + ferrosilicon” system, Ea increases to 37.64 kJ·mol^− 1^ at T = 480–560 °C, while for the “ore + aluminum slag” system, Ea reaches 50.20 kJ·mol⁻¹ at T = 560–620 °C. The resulting sequence of reactivity (ferrosilicoaluminum < ferrosilicon < aluminum slag) indicates a pronounced synergistic effect of the combined presence of silicon and aluminum, which reduces the energy barriers of reduction reactions and enhances the kinetic activity of the system.

**Determination of the Activation Energy of Thermal Transformations**.

The activation energy of thermal transformations was evaluated based on non-isothermal differential thermal analysis (DTA) data^31^.

The calculation is based on the Arrhenius equation, which relates the reaction rate constant *k* to temperature:

$$\:k=Aexp\left(-\frac{{E}_{a}}{RT}\right)$$

where *A* is the pre-exponential factor, *Ea* is the activation energy, *R* = 8.314 J·mol^− 1^·K^− 1^ is the universal gas constant, and *T* is the absolute temperature (K).

As a first approximation, the deviation of the DTA signal from the baseline, Δt, is assumed to be proportional to the rate of the thermal process *k*. Under this assumption, the Arrhenius equation can be written in logarithmic form:

$$\:\mathrm{l}\mathrm{n}\left(\varDelta\:t\right)=lnA-\frac{{E}_{a}}{RT}$$

or, when expressed in base-10 logarithms:

$$\:lg\left(\varDelta\:t\right)=lgA-\frac{{E_a}}{R}\cdot\:\frac{1}{T}$$

Graphical plotting of the dependence $\:\mathrm{l}\mathrm{g}\left({\Delta\:}t\right)=f(1/T)$allows a linear approximation to be obtained, the slope of which is given by:

$$\:{k}_{\alpha\:}=\frac{dlg\left(\varDelta\:t\right)}{d\left(\frac{1}{T}\right)}=-\frac{{E_a}}{{2303}\cdot R}$$

From this relationship, the activation energy is calculated as:

$$\:{E}_{akm}=-{2303}\:R\cdot{k}_{\alpha\:}$$

Thus, the activation energy is determined as a quantity proportional to the slope of the linear approximation of the dependence of $\:\mathrm{l}\mathrm{g}\left({\Delta\:}t\right)$on $\:1/T$, obtained for individual stages of thermal decomposition or phase transformations.

The values of *T*, DTG (= Δt), lg(DTG) (= lg Δt), and 1/*T* used for calculating the activation energy for each investigated material are presented in Table 5.

**Table 5:** Values of T, DTG, lg(DTG), and 1/T used for activation energy calculations.

Peak No.	Т, °С	Т, К	*Δt*, %/min	lg *Δt*	1/T·10^3^
**Nickel ore**					
1	360	633	0.20	−0.699	1.58
	380	653	0.25	−0.602	1.53
	407	680	0.27	−0.569	1.47
	439	712	0.32	−0.495	1.40
2	507	780	0.25	−0.602	1.28
	527	800	0.30	−0.523	1.25
	538	811	0.36	−0.444	1.23
	564	837	0.41	−0.387	1.19
3	760	1033	0.12	−0.921	0.97
	775	1048	0.18	−0.745	0.95
	790	1063	0.21	−0.678	0.94
	805	1078	0.24	−0.620	0.93
**Mixture of nickel ore with aluminum slag**					
1	560	833	0.29	−0.538	1.20
	576	849	0.32	−0.495	1.18
	590	863	0.39	−0.409	1.16
	608	881	0.42	−0.377	1.14
	620	893	0.47	−0.328	1.12
**Mixture of nickel ore with ferrosilicon**					
1	560	833	0.29	−0.538	1.20
	576	849	0.32	−0.495	1.18
	590	863	0.39	−0.409	1.16
	608	881	0.42	−0.377	1.14
	620	893	0.47	−0.328	1.12
	560	833	0.29	−0.538	1.20
**Mixture of nickel ore with ferrosilicoaluminum**					
1	527	800	0.234	−0.631	1.25
	555	828	0.258	−0.588	1.21
	580	853	0.280	−0.553	1.17
	605	878	0.298	−0.526	1.14
	625	898	0.302	−0.520	1.11

It should be noted that the obtained activation energy values are approximate and comparative in nature and are used to compare the kinetic features of thermal processes occurring in the investigated systems.

Thus, the ferrosilicoaluminum-based system exhibits the lowest activation energy (16.15 kJ·mol^− 1^), which is 2.3 times lower than that of the ferrosilicon system (37.64 kJ·mol^− 1^) and 3.1 times lower than that of the aluminum slag system (50.20 kJ·mol^− 1^). This provides direct experimental evidence of a synergistic effect arising from the combined use of silicon and aluminum within a single reducing agent. The low Ea value indicates higher reactivity and reduced kinetic limitations, enabling the process to proceed efficiently at lower temperatures.

### Process optimization using design of experiments

To quantitatively assess the influence of the main factors and to identify optimal process conditions, a second-order rotatable central composite design (CCD) was applied^33,34^. The design matrix and the results of thermodynamic modeling performed in FactSage are presented in Table 6.

**Fig. 11:**
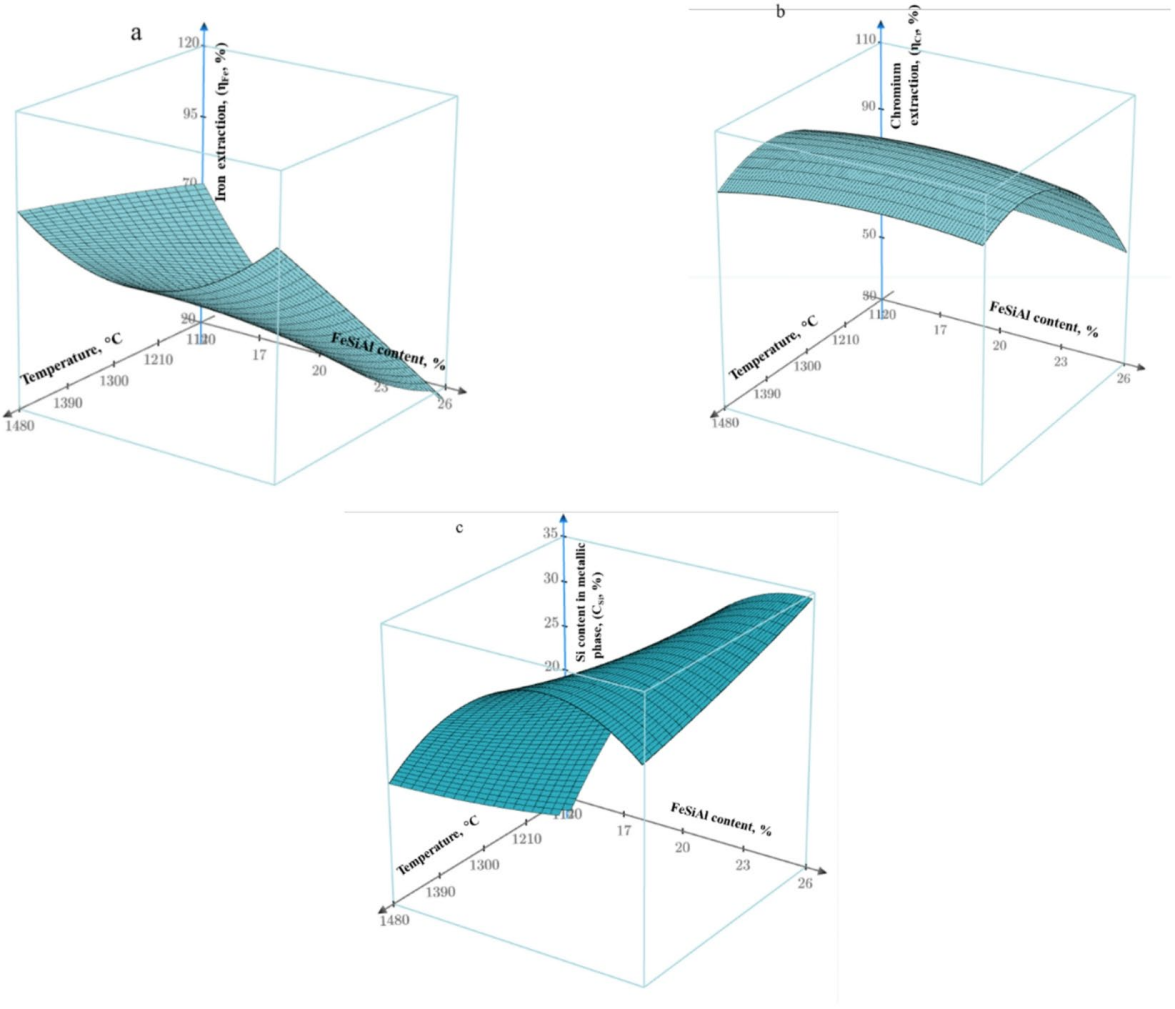
Response surfaces: (**a**) degree of iron reduction (ηFe); (**b**) degree of chromium reduction (ηCr); (**c**) silicon content in the metallic phase (CSi) as a function of temperature and ferrosilicoaluminum content.

Analysis of the response surfaces (Fig. 11) shows that increasing temperature has a positive effect on the values of ηFe and ηCr, whereas an increase in the ferrosilicoaluminum content results in a nonlinear response. It was established that increasing the proportion of ferrosilicoaluminum enhances the recovery of iron and chromium; however, it simultaneously leads to an increase in the silicon concentration in the metallic phase, as confirmed by the response surface shown in Fig. 11c. The calculations indicate that an increase in the ferrosilicoaluminum content promotes intensified transfer of silicon into the metallic phase, while the contribution of aluminum is mainly realized through modification of the slag composition and properties. Accordingly, the optimal operating conditions correspond to a temperature of 1300–1350 °C, a ferrosilicoaluminum content of 10 wt%, and a lime addition of 38–40 wt%.

The ηNi value was equal to 100% at all points of the design matrix, showing no variation within the investigated factor ranges. Therefore, ηNi was not considered as a response variable in the regression modeling.

Based on the processing of thermodynamic data, second-order regression equations were obtained for the degree of iron reduction, the degree of chromium reduction, and the silicon content in the metallic phase. These models were used to construct three-dimensional response surfaces, where X_1_, X_2_, and X_3_ represent the coded values of temperature, reducing agent content, and lime content, respectively.

**Table 6:** Experimental design matrix.

No.	X₁ (T, °C)	X_2_ (FeSiAl content, wt%)	X_3_ (lime content, wt%)	ηFe, %	ηCr, %	CSi, %
1	1121.6	14.05	21.08	80.31	67.85	17.87
2	1478.4	14.05	21.08	80.29	66.28	17.95
3	1121.6	25.95	21.08	2.98	32.40	38.27
4	1478.4	25.95	21.08	86.31	66.37	33.85
5	1121.6	14.05	38.92	64.75	95.85	20.03
6	1478.4	14.05	38.92	99.95	99.94	24.91
7	1121.6	25.95	38.92	30.38	99.99	48.81
8	1478.4	25.95	38.92	99.98	99.99	36.97
9	1000	20.00	30.00	8.64	14.97	19.17
10	1300	10.00	30.00	99.97	99.93	30.45
11	1300	30.00	30.00	99.94	98.73	11.23
12	1300	20.00	30.00	63.23	99.98	42.56
13	1300	20.00	15.00	99.96	99.63	25.33
14	1300	20.00	45.00	52.82	99.99	36.07
15	1300	20.00	30.00	45.39	99.92	27.00
16	1300	20.00	30.00	45.37	99.92	26.99
17	1300	20.00	30.00	45.38	99.92	27.00
18	1300	20.00	30.00	45.38	99.92	27.00
19	1300	20.00	30.00	45.38	99.93	27.00
20	1300	20.00	30.00	45.38	99.93	27.00

The calculations were performed at a fixed temperature. After initiating the computation, the equilibrium distribution of iron (or chromium) between the slag and metallic phases was determined. The recovery of iron (ηFe) and chromium (ηCr) was calculated as the ratio of the amount of iron or chromium transferred into the metallic phase to its initial content in the charged feed:

$$\:{\eta\:}_{Fe}=\frac{{Fe}_{metal}}{{Fe}_{initial}}\:\cdot100\%;\:{\eta\:}_{Cr}=\frac{{Cr}_{metal}}{{Cr}_{initial}}\:\cdot100\%$$

The amount of iron and chromium transferred to the metallic phase (*Fe*_*metal*_), (*Cr*_*metal*_) was determined using the following equation:

$$\:{Fe}_{metal}={m}_{metal}\cdot\frac{{w}_{Fe,\:metal}}{100}\:;\:{Cr}_{metal}={m}_{metal}\cdot\frac{{w}_{Cr,\:metal}}{100}$$

where *m*_*metal*_ is the mass of the metallic phase (g), and *w*_*Fe, metal, Cr, metal*_ are the mass fractions of iron and chromium in the metal, respectively.

The amounts of iron and chromium in the charge material were determined using the following equation:

$$\:{Fe}_{initial}={(m}_{ore}\cdot\frac{{w}_{Fe\left(ore\right)}}{100})+\left({m}_{FeSiAl}\cdot\frac{{w}_{Fe}}{100}\right);{Cr}_{initial}={(m}_{ore}\cdot\frac{{w}_{Cr\left(ore\right)}}{100})+({m}_{FeSiAl}\cdot\frac{{w}_{Cr}}{100})$$

where *m*_*ore*_ is the mass of the ore (g); *w*_*Fe(ore)/Cr(ore)*_ are the mass fractions of iron/chromium in the ore (wt%); *m*_*FeSiAl*_ is the mass of the reducing agent (g); and *w*_*Fe/Сr*_ are the mass fractions of iron/chromium in the reducing agent (wt%).

Based on the data of the design matrix presented in Table 6, the results were mathematically processed using regression analysis for a second-order rotatable central composite design. As a result of the experimental design analysis, a second-order regression model describing the dependence of the iron reduction degree on the investigated factors was developed.

Using this regression model, a three-dimensional response surface was constructed in the MathCad software environment. When generating the response surface, the value of factor X₃ (lime content) was fixed at zero, corresponding to 30 wt% lime in the charge. According to the results of the second-order rotatable design, regression models in the form of second-order polynomials were obtained for each response variable. These models served as the basis for constructing the response surfaces presented in the “Results” section.

### Validation of the optimized regime by large-scale laboratory smelting

Based on the results of the second-order rotatable central composite design and the analysis of the response surfaces, laboratory smelting experiments of a nickel-containing alloy were carried out to validate the calculated data. Taking into account the identified optimal region of process parameters, three charge compositions were selected for experimental verification (Table 7).

**Table 7:** Composition of charge materials for large-scale laboratory tests.

FeSiAl content, wt%	Lime content, wt%
10	30
20	30
30	30

Nickel ore with the chemical composition given above in Table 1 (Sample 1) was used as the raw material, along with ferrosilicoaluminum containing (wt%): 44.7 Fe, 40.0 Si, 15.0 Al, 0.2 C, and 0.05 P, and lime containing 90 wt% CaO.

The validation experiments were carried out using Sample 1, which contains higher concentrations of nickel (1.18% vs. 0.82%) and iron (20.5% vs. 10.8%) and is therefore considered more technologically representative for assessing the efficiency of metallothermic reduction and the reliability of the optimized process parameters.

The applicability of the optimized regime to Sample 2 requires additional experimental verification. However, based on the physicochemical characteristics obtained in this study, several tendencies may be expected. The higher contents of Al_2_O_3_ (12.5%) and MgO (7.7%) in Sample 2 are expected to increase the slag liquidus temperature and viscosity, which may require a higher lime addition (42–45 wt%) to ensure adequate slag fluidity. At the same time, the lower softening end temperature of Sample 2 (1281 °C compared with 1376 °C for Sample 1) suggests the possibility of operating at slightly lower temperatures (1250–1300 °C).

Therefore, the optimized regime determined in this study (1300–1350 °C, 10 wt% FeSiAl) may be considered as an initial reference point for process optimization for Sample 2, although the lime addition should be adjusted accordingly.

The FeO/NiO ratio in the ore was approximately 17.2. At FeO/NiO ratios exceeding 15, only low-grade ferronickel (less than 8 wt% Ni) can be produced^35^, which has a very limited market demand. To expand the potential application range of low-grade ferronickel, it was proposed to broaden the alloy composition by achieving deeper reduction of components from nickel-bearing melts.

**Fig. 12:**
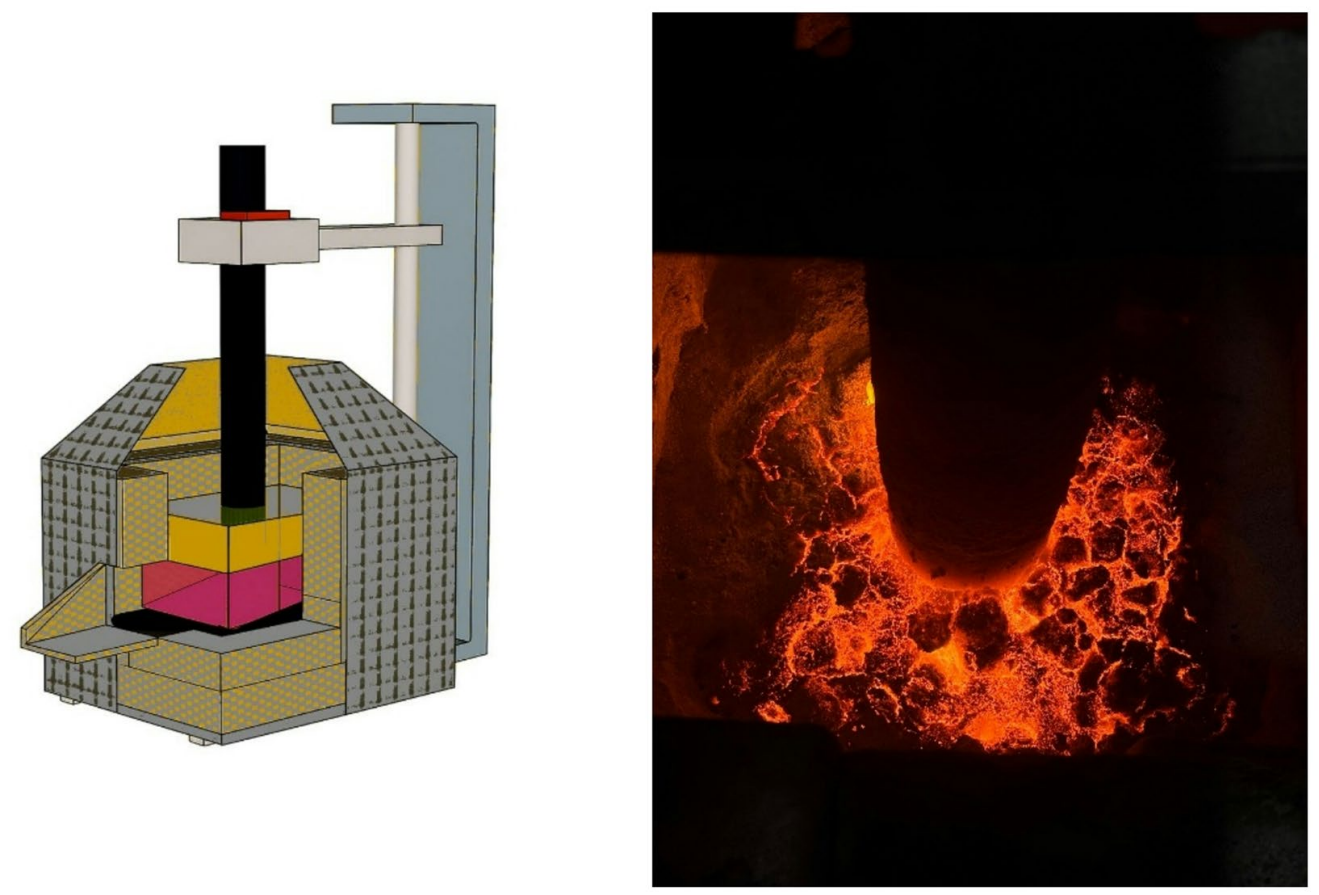
General view of the laboratory ore-thermal electric furnace and the arc-heating zone of the melt.

The experiments were carried out in a single-phase electric furnace with a transformer power of 100 kVA and an electrode diameter of 100 mm. The furnace transformer had three secondary voltage levels: 12, 18, and 24 V. Smelting was conducted at a secondary voltage of 18 V. The current on the high-voltage side of the transformer ranged from 80 to 100 A. A general view of the furnace is shown in Fig. 12.

The technological procedure of electric smelting was carried out as follows. Preheating of the electric furnace was performed for 120 min using coke fines as an electrical current conductor in an amount of 0.2 kg. After completion of the preheating stage, the furnace bath was completely cleaned of residual coke fines to enable subsequent charging of the main burden. Following preheating, 0.5 kg of nickel ore was charged onto the furnace hearth to form an initial melt. The particle size of the charge materials ranged from + 5 to − 10 mm.

After melt formation and stabilization of the electrical load, a full burden charge was introduced, consisting of 9.5 kg of nickel ore (0.5 kg having been consumed at the initial stage), 1.0 kg of ferrosilicoaluminum reducing agent, and 3.0 kg of lime. The consumption of charge materials per smelting run corresponded to one burden of the above composition. Temperature control was performed indirectly, based on the melting behavior of the burden layer, the stability of the arc regime, and periodic tapping of metal and slag at intervals of every 2 h. The tap hole was opened using a steel rod, and the melt flowed actively. Good separation between metal and slag was observed.

The second charge consisted of 10 kg of nickel ore, 2.0 kg of reducing agent, and 3.0 kg of lime. The smelting procedure was carried out in the same manner as for the first charge. During smelting, no significant deviations from the normal technological regime were observed. Metal and slag were tapped actively. When the reducing agent content was increased to 3.0 kg of ferrosilicoaluminum, the melt in the burden zone became viscous; therefore, an additional 0.5 kg of lime was added.

**Fig. 13:**
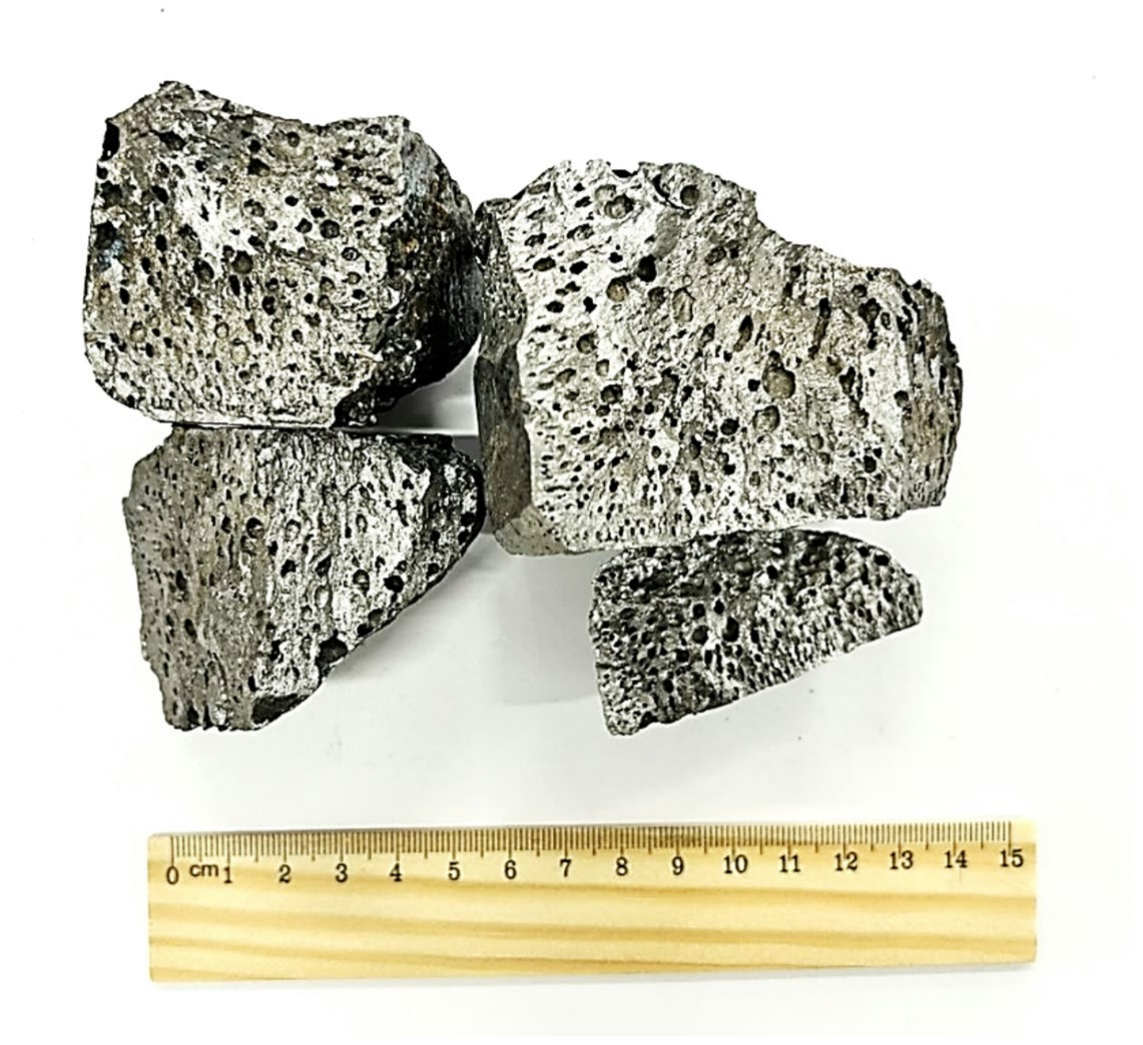
External appearance of the nickel-containing alloy.

Overall, the campaign yielded 9.5 kg of alloy, 26.2 kg of slag, and a residual solid mass in the furnace (“skull”) of 3.0 kg. As a result of the experiment, an alloy with the following composition (wt%) was obtained: 70.0 Fe, 8.0 Ni, 17.0 Si, 3.5 Cr, and 0.8 Al (Fig. 13), along with a slag containing (wt%): 48.6 SiO_2_, 36.4 CaO, 10.2 Al_2_O_3_, 4.5 MgO, and 0.1 NiO. Below, a comparison is presented between the iron recovery (ηFe), chromium recovery (ηCr), and silicon concentration in the metal (CSi) obtained by thermodynamic modeling and experimental investigations (Table 8).

**Table 8:** Comparison of thermodynamic modeling and experimental results for metal recovery and silicon content.

Parameter	Thermodynamic modeling, %	Experiment, %	Deviation, %
η_Fe_	62.1	71.0	−12.5
η_Cr_	87.1	83.0	4.9
η_Ni_	100.0	100.0	0
C_Si_	20.0	17.0	17.6

To assess reproducibility, the validation smelting at the optimal parameters was repeated three times. The standard deviations for ηFe, ηCr, and CSi were ± 2.8%, ± 1.5%, and ± 0.6%, respectively, indicating good reproducibility of the experimental procedure.

The observed discrepancy between the modeled (62.1%) and experimental (71.0%) iron recovery (+ 12.5%) can be attributed to:

1. Kinetic limitations in the model: FactSage calculates equilibrium, which may not be fully reached in practice; local superheating and intense mixing in the arc zone can enhance reduction beyond equilibrium predictions.
2. Incomplete phase separation: mechanical entrainment of fine metal droplets in slag or slag emulsification in metal can affect analytical results.
3. Local compositional variations: imperfect homogenization may create micro-volumes with higher reducing potential.
4. Cumulative analytical uncertainties. Overall, a deviation of 12.5% is acceptable at laboratory scale and confirms the model’s adequacy for predicting trends and process optimization.

The experimental smelting tests confirmed the fundamental feasibility of the selected operating regime and ensured the production of a nickel-containing alloy with stable metal–slag separation.

Detailed phase and microstructural analysis of the obtained alloy was beyond the scope of this study, as the primary goal was to prove the feasibility of producing a Ni-Cr-Si alloy by metallothermy and to optimize the smelting parameters. Future work will focus on characterizing the phase composition (by XRD, SEM-EDS) and properties (microhardness, melting range) of the alloy to substantiate its application as a master alloy.

The obtained alloy contains 17–18 wt% Si, which is higher than that of conventional ferronickel. However, this feature is not considered a drawback but rather a technological characteristic of the proposed metallothermic process. The elevated silicon content enables the alloy to function as an effective complex deoxidizing and alloying additive in steelmaking. When introduced into liquid steel, silicon acts as a deoxidizer, while nickel and chromium simultaneously contribute to alloying, which may potentially reduce the need for separate additions of ferrosilicon and ferronickel.

At the same time, the relatively high silicon content may limit the direct use of the alloy in steel grades requiring very low silicon levels. Therefore, the intended application of the material is as a complex master alloy, rather than as a direct substitute for conventional ferronickel. If necessary, the silicon content can be reduced by decreasing the proportion of FeSiAl in the charge; however, this adjustment may lead to a decrease in the recovery of iron and chromium during the smelting process.

## Conclusion

Thermodynamic analysis performed using HSC Chemistry confirmed that the combined use of silicon and aluminum (the NiO-Si-Al system) provides the most favorable conditions for nickel reduction compared with the separate application of these reducing agents. This is evidenced by more negative ΔG⁰ values over the entire investigated temperature range of 100–1600 °C.

Kinetic analysis based on TG-DTA data revealed the key advantage of the complex reducing agent ferrosilicoaluminum (FeSiAl). The calculated apparent activation energy (Ea) for the “ore-FeSiAl” system is 16.15 kJ·mol^− 1^, which is 2.3 and 3.1 times lower than those for the systems with ferrosilicon (37.64 kJ·mol^− 1^) and aluminum slag (50.20 kJ·mol^− 1^), respectively. This provides direct evidence of a pronounced synergistic effect that significantly lowers the kinetic barrier of reduction reactions.

The application of design of experiments methods (rotatable central composite design) combined with thermodynamic modeling in FactSage made it possible to determine the optimal smelting regime for producing a nickel-containing alloy from lateritic ore: a temperature of 1300–1350 °C, an FeSiAl content of 10 wt%, and a lime content of 38–40 wt%. Under these conditions, maximum recovery of iron and chromium is achieved (ηFe = 62.1%, ηCr = 87.1%, ηNi = 100% according to the model) while maintaining a controlled silicon content in the metal (CSi ≈ 20 wt%).

Validation laboratory smelting experiments confirmed the effectiveness of the optimized regime. A total of 9.5 kg of a multicomponent alloy with the following composition (wt%) was obtained: Fe 70.0, Ni 8.0, Si 18.0, Cr 5.0, and Al 0.8. The recoveries into the alloy were ηFe = 71%, ηCr = 83%, and ηNi = 100%. The low residual NiO content in the slag (0.1 wt%) indicates a high degree of nickel reduction. The Fe/Ni ratio exceeding 15 in the initial ore predetermined the production of not a conventional ferronickel, but a new multifunctional Fe-Ni-Si-Cr-Al alloy.

The proposed FeSiAl-based metallothermic process represents an energy-efficient and environmentally more sustainable alternative to conventional carbothermic technology.

Preliminary assessments suggest that the proposed metallothermic process may offer significant environmental advantages. In particular, direct CO_2_ emissions during the smelting stage are estimated to decrease by approximately 85–90%, as the use of carbonaceous reducing agents is largely eliminated (the FeSiAl reducing agent contains only about 0.2 wt% C).

In addition, the process is carried out at 1300–1350 °C, which is 50–100 °C lower than the temperatures typically required for conventional carbothermic smelting (> 1400 °C), potentially resulting in energy savings of approximately 10–15%.

At the same time, it should be noted that the production of FeSiAl is itself energy intensive. Therefore, a comprehensive cradle-to-gate life cycle assessment (LCA) is required to quantitatively evaluate the overall environmental impact of the proposed technology. Such an analysis is planned for future work.

Owing to the combined presence of nickel, silicon, and chromium, the produced alloy can be used as a complex alloying additive and deoxidizer in the production of corrosion-resistant steels or as a reducing agent in metallurgical processes.

The comprehensive approach integrating thermodynamic modeling, kinetic analysis, experimental design, and practical validation has demonstrated the high efficiency of ferrosilicoaluminum as a complex reducing agent for producing nickel-containing alloys from lateritic ores.

## Data Availability

All data and materials were provided in the manuscript and readers can contact to corresponding author to receive additional explanation.
